# Aneurysmal bone cysts (ABC): Retrospective analysis of two hundred and fifty eight cases

**DOI:** 10.1007/s00264-025-06603-3

**Published:** 2025-07-11

**Authors:** Mustafa Onur Karaca, Orhun Eray Bozkurt, Merve Dursun Savran, Mustafa Özyıldıran, Kerem Başarır, Hüseyin Yusuf Yıldız

**Affiliations:** 1 Ankara University Faculty of Medicine, Orthopedics and Traumatology Department, Ankara, Turkey; 2 Kulu Region State Hospital, Orthopedics and Traumatology Department, Konya, Turkey; 3 Sandıklı State Hospital, Orthopedics and Traumatology Department, Afyon, Turkey; 4 Private Practice, Orthopedics and Traumatology, Ankara, Turkey

**Keywords:** Aneurysmal bone cyst, Recurrence, Electrocautery, Burr, Curettage

## Abstract

**Purpose:**

Aneurysmal bone cysts (ABCs) are bone tumours characterised by blood-filled cystic lesions. Management strategies for ABCs vary widely and lack consensus. This study aims to evaluate outcomes in 258 patients and investigate the factors affecting the recurrence rates.

**Methods:**

This study is a single-centre retrospective analysis of patients diagnosed with ABC between January 1990 and December 2020. Patients who were histologically diagnosed with ABC, had available pathology, radiology, and surgery records, and were followed up for at least 24 months were included. Secondary ABCs were excluded. Presenting symptoms and location, computerised tomography (CT) and magnetic resonance imaging (MRI), treatment modalities, and recurrence were investigated.

**Results:**

The mean age of the 258 ABC patients was 17.25 ± 12.37 years, 67.44% being under 18 years, and 12.40% under five years. 49.45% were female. The average follow-up duration was 47.80 ± 41.92 months. Pain was the most common presenting symptom, reported by 79.97% of patients. 5.04% were asymptomatic and diagnosed incidentally, whereas 11.63% were diagnosed following a pathological fracture. The median disease-free survival was ten months, with the average time to first recurrence being 24.22 ± 22.14 months. Recurrence was more common in patients under five years of age (34.38% vs. 19.03%, *p* = 0.046) and in those with pathologic fractures (40.00% vs. 18.42%, *p* = 0.006). Conversely, recurrence was less common when burr and/or cautery was added to curettage (31.97% vs. 11.03%, *p* < 0.001). Time to recurrence was significantly shorter in cases with soft tissue oedema (median 5 vs. 12 months, *p* = 0.010) or fluid-fluid levels (median 6 vs. 12 months, *p* = 0.038).

**Conclusions:**

The study found that pathological fractures and age under five years are associated with a higher risk of recurrence in aneurysmal bone cysts. Electrocauterization and/or high-speed burring as local adjuvant therapy is associated with low recurrence rates.

## Introduction

Aneurysmal bone cysts (ABCs) represent a classification of bone tumours distinguished by a cystic lesion within the bone, replete with sanguineous content. Though commonly found in the metaphysis of long bones and vertebrae, ABCs can occur in any bony structure throughout the body. The yearly occurrence rate of ABCs is approximated at 0.14 per 100,000 individuals, constituting a 1–6% prevalence among all primary, benign bone neoplasms [1–3]. ABCs can develop at any age but are notably more prevalent in individuals under 20, accounting for 75–90% of cases within this age group [4, 5].

Clinically, patients with ABCs present with pain, which may or may not be accompanied by noticeable swelling. In some cases, the initial presentation may include a pathological fracture at the lesion site [6, 7]. Radiographically, ABCs are identified by their well-defined, expansile nature with prominent trabeculations [4, 8]. While some cysts may spontaneously resolve or show resolution following biopsy, the standard treatment approach remains intralesional curettage, potentially combined with bone grafting. Management strategies vary widely and may include embolization, curettage with or without bone grafting, cavity cementation, reconstructive surgery, and, more recently, sclerotherapy [4, 7, 9–15].

Despite the availability of these treatment options, a consensus on the optimal management strategy for ABCs remains elusive. It is known that postoperative recurrence is not uncommon, and the rates of recurrence are reported to range from 3 to 59% in the literature [4, 16–18].

There are several critical questions: Are there any radiological findings that predict recurrence? How do age, physeal status, and related factors affect recurrence? How does the use of adjuvant treatments, such as cauterization and burring, impact recurrence?

This study aims to address these questions by evaluating the experiences of 258 patients within our clinical setting and assessing the impact of adjuvant burring and cauterization in conjunction with curettage on the recurrence of ABCs.

## Methods

The study protocol was approved by the University Medical Faculty Human Studies Institutional Ethics Review Board (ID: İ04-362-24).

**Fig. 1:**
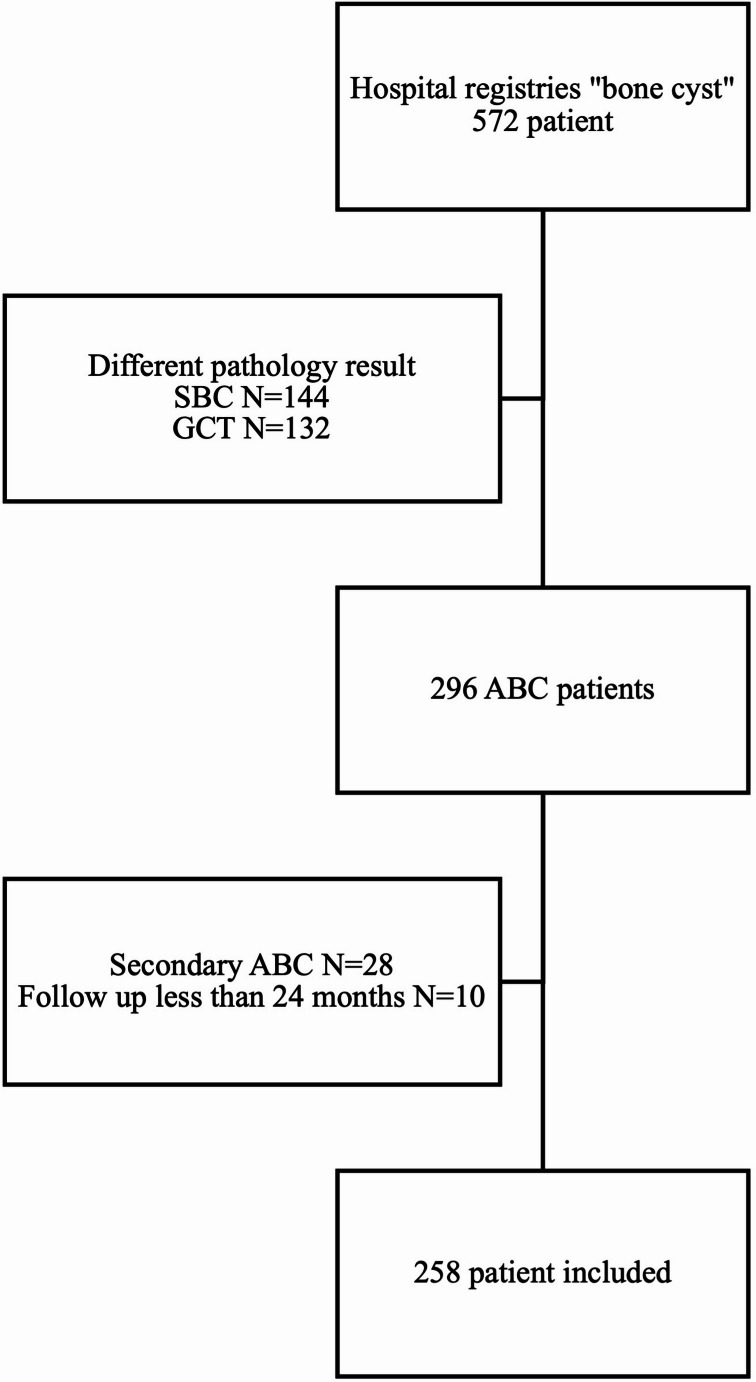
Excluded and included patients

This study is a single-centre retrospective study. All patients diagnosed as ABC at the University Hospital between January 1990 and December 2020 were investigated. All patients histologically diagnosed as ABC, whose pathology, radiology, and surgery records are available and followed up at least 24 months, were included. From the hospital registries, 572 patients were found for the search “bone cyst.” 144 patients with simple bone cysts (SBC) and 132 patients with giant cell tumours (GCT) were excluded. From the remaining 296 ABCs, 28 were secondary, and ten had less than 24 months of follow-up. So, they were excluded, and only 258 ABCs were included (Fig. 1).

The differential diagnosis of aneurysmal bone cysts includes benign lesions as well as malignant tumours, such as telangiectatic osteosarcoma. Accurate histological diagnosis is essential, as the prognosis and treatment of ABCs are different from those of malignant bone lesions [7, 19]. Therefore, patients underwent either core needle biopsy or incisional biopsy preoperatively. All patients whose diagnosis was verified histologically according to the pathology report were included in the study.

Presenting symptoms and location from the initial outpatient clinic notes were recorded. Imaging studies, such as radiographs, computerised tomography (CT) and magnetic resonance imaging (MRI) were investigated. The presence of pathologic fracture, bone destruction, cortical reaction, and periosteal reaction was mainly evaluated using X-rays and CT. On the other hand, bone marrow and soft tissue oedema and fluid-fluid levels were assessed by MRI. The Enneking staging system was used to perform tumour staging. Preoperative radiographs were evaluated and the lesions were classified as inactive, active, or aggressive. Lesions with well-defined rims and confined to the bone were classified as inactive. Lesions that were confined to the bone with incomplete reactive rims were classified as active lesions. Tumours with a poorly defined margin were categorized as aggressive. Imaging interpretations for musculoskeletal tumours are routinely performed by a radiologist specialised in musculoskeletal imaging at the institution. For this study, both the interpretations provided by this radiologist and the results reviewed by an orthopaedic specialist with expertise in musculoskeletal oncology were recorded.

Treatment modalities, including curettage, burr, and cauterization, were extracted, and documented from the surgical operation reports. The presence of recurrence was investigated by examining postoperative radiographic images and medical records. Cases with a persistent radiolucent lesion that showed radiographic progression on follow-up radiographs were considered recurrences. Patients were contacted via telephone for missing data not found in the patient records.

StataMP13 (StataCorp. Stata Statistical Software: Release 13) was used for descriptive and inferential analyses. Shapiro-Wilk test was used to assess normality. Chi-square and Fisher’s exact tests were used for categorical variables. A T-test was performed to analyse the parametric data between groups. Mann Whitney U test was performed to analyse the non-parametric data between groups. Wilcoxon test was used to analyse data between dependent groups (pre-post comparison). ANOVA test followed by post hoc analysis was performed to analyse the parametric data between multiple groups. Kruskal Wallis test, followed by post hoc analysis, was performed to analyse the non-parametric data between multiple groups. A p-value less than 0.05 was accepted as significant.

## Results

The mean age of 258 ABC patients was 17.25 ± 12.37. 67.44% were younger than 18, and 12.40% were younger than five. 49.45% were women and 50.55% were men. The average follow-up was 47.80 ± 41.92 months.

**Fig. 2:**
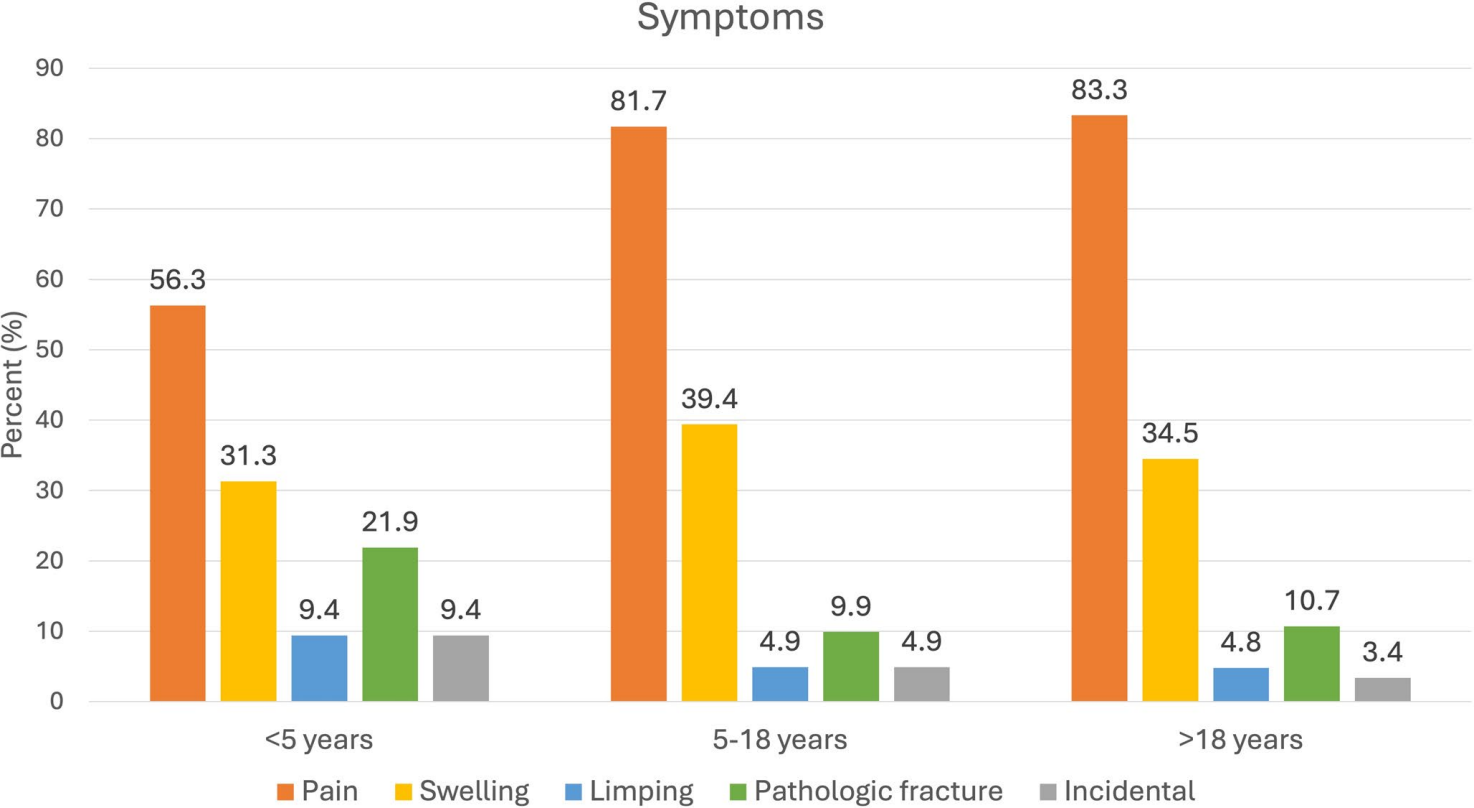
Symptomatology in different age groups

The most common presenting symptom was pain (79.97%), followed by swelling (36.82%). Pain was less common if the patient was younger than five years (*p* = 0.003). 13 patients (5.04%) had no symptoms and were diagnosed incidentally, whereas 30 patients (11.63%) were diagnosed after a pathological fracture. Presenting symptoms according to age groups are given in Fig. 2. The mean duration of symptoms before the presentation was 7.77 ± 19.48 months.

**Fig. 3:**
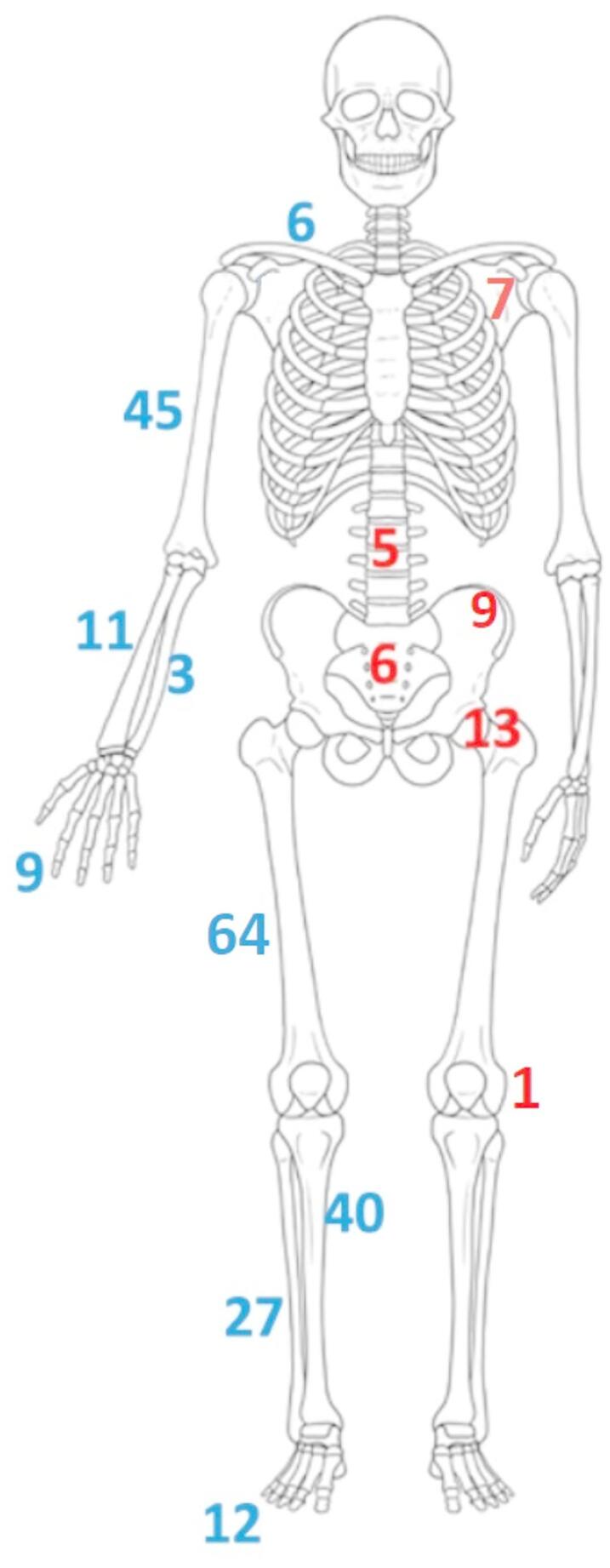
Anatomical locations of the lesions

15.89% of the ABCs were located in the spine and flat bones, whereas 84.11% were in long bones. There was no difference in recurrence rates between flat bones and long bones (*p* = 0.134). Detailed distribution of ABCs along the body is given in Fig. 3. The most common bones were the femur, humerus, and tibia. 32.26% ABSs were meta-diaphyseal, 23.96% were metaphyseal, 20.74% were meta-epiphyseal, 17.51% were diaphyseal, and only 5.53% were epiphyseal.

**Fig. 4:**
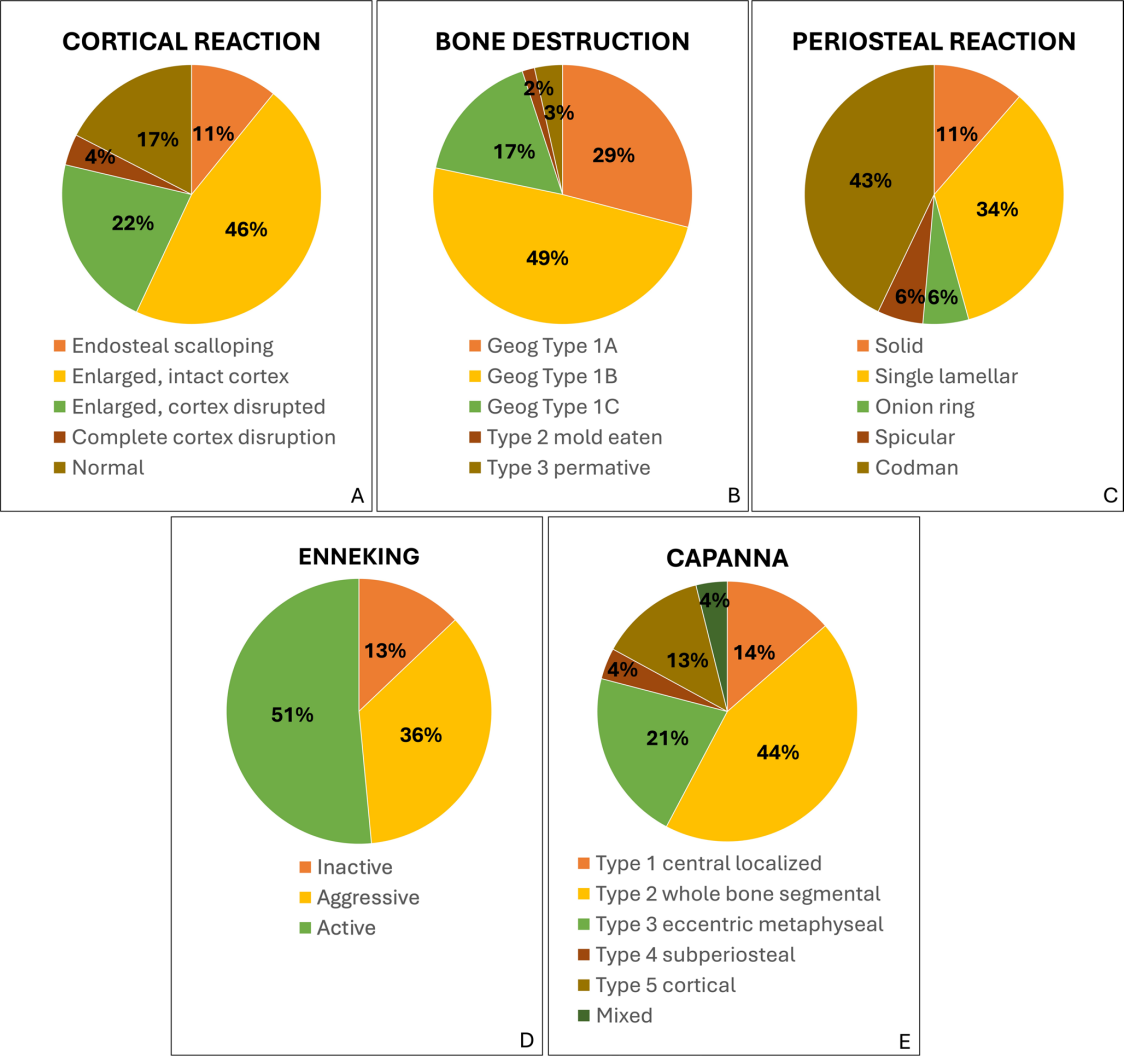
Distribution of cortical reaction (**A**), bone destruction (**B**), and periosteal reaction (**C**) type, Enneking (**D**) and Capanna (**E**) classifications

The distribution of cortical reactions, periosteal reactions, and bone destruction were given in Fig. 4 (A-C). The most common type of bone destruction was geographical type IB, and the most common type of cortical reaction was enlarged, intact cortex type. Shell formation was present in 48.4%, 24.3% were partial, and 24.1% were complete. According to MRI findings, soft tissue edema was present in 29 (11.24%) cases, bone marrow oedema in 38 (14.73%) cases, and a fluid-fluid level in 178 (68.99%) cases.

According to the Enneking staging system, the lesions were inactive in 33 (12.79%) cases, active in 134 (51.94%) cases, and aggressive in 91 (35.27%) cases. According to Capanna classification, 35 (13.56%) lesions were type I, 114 (44.18%) were type II, 55 (21.31%) were type III, 10 (3.87%) were type IV, and 34 (13.18%) were type V, and ten (3.87%) were mixed type. The relationship between the Enneking stage and Capanna morphological subgroups with recurrence was also investigated. The recurrence rate was not found to be statistically associated with these classification systems. The Enneking and Capanna distributions are presented in Table 1; Fig. 4 (D, E).

**Table 1:** Factors affecting recurrence and time to recurrence

		Recurrence *N* = 54	No recurrence *N* = 204	*P* value for recurrence	Survival analysis for recurrence
**Age**	Mean ± standard deviation	15.94 ± 12.95	17.59 ± 12.22	0.1249	-
	< 18 > 18	36 (20.69%) 18 (21.43%)	138 (79.31%) 66 (78.57%)	0.891	0.184
	< 5 > 5	11 (34.38%) 43 (19.03%)	21 (65.62%) 183 (80.97%)	**0.046**	0.741
**Presentation**	Pathologic fracture + No fracture	12 (40.00%) 42 (18.42%)	18 (60.00%) 186 (81.58%)	**0.006**	0.203
**Localization**	Flat bones and spine Long bones	5 (12.20%) 49 (22.58%)	36 (87.80%) 168 (77.42%)	0.134	0.331
	Epiphysis Metaepiphysis Metaphysis Metadiaphysis Diaphysis	3 (25.00%) 11 (24.44%) 10 (19.23%) 21 (30.00%) 4 (10.52%)	9 (75.00%) 34 (75.55%) 42 (80.76%) 49 (70.00%) 34 (89.47%)	0.206	0.793
**Joint and physis relation**	Physis open Physis closed	36 (22.22%) 18 (18.75%)	126 (77.78%) 78 (81.25%)	0.508	0.375
	Adjacent to physis * Non-adjacent to physis *	20 (23.26%) 16 (21.05%)	66 (76.78%) 60 (78.95%)	0.736	0.697
	Adjacent to joint Non-adjacent to joint	14 (18.42%) 40 (22.47%)	62 (81.58%) 138 (77.53%)	0.470	0.117
**Bone destruction**	Geographical Type IA Geographical Type IB Geographical Type IC Type II moth eaten Type III permeative	13 (17.33%) 30 (23.62%) 10 (23.26%) 0 (0%) 1 (11.11%)	62 (82.67%) 97 (76.38%) 33 (76.74%) 4 (100%) 8 (88.89%)	0.580	0.531
**Cortical reaction**	Endosteal scalloping Enlarged, cortex intact Enlarged, cortex destructed Complete destruction Normal	5 (17.86%) 24 (20.17%) 12 (21.43%) 1 (10.00%) 12 (26.67%)	23 (82.14%) 95 (79.83%) 44 (78.57%) 9 (90.00%) 33 (73.33%)	0.768	0.536
**Periosteal reaction**	Solid Single lamellar Onion ring Spiculated Sunburst Codman	0 (0%) 4 (33.33%) 0 (0%) 1 (50.00%) - 3 (20.00%)	4 (100%) 8 (66.67%) 2 (100%) 1 (50.00%) - 12 (80.00%)	0.489	0.947
**MRI finding**	Soft tissue edema No soft tissue edema	6 (20.69%) 48 (20.96%)	23 (79.31%) 181 (79.04%)	0.973	**0.010**
	Fluid– fluid level No fluid– fluid level	39 (21.91%) 15 (18.75%)	139 (78.09%) 65 (81.25%)	0.564	**0.038**
**Enneking**	Inactive Active Aggressive	7 (21.21%) 22 (16.42%) 25 (27.47%)	26 (78.79%) 112 (83.58%) 66 (71.53%)	0.135	0.612
**Capanna**	Type I central localized Type II whole segment Type III eccentric metaphysis Type IV subperiosteal Type V cortical Mixed	4 (11.43%) 30 (26.32%) 12 (21.82%) 3 (30.00%) 3 (8.82%) 2 (20.00%)	31 (88.57%) 84 (73.68%) 43 (78.18%) 7 (70.00%) 31 (91.18%) 8 (80.00%)	0.189	0.514
**Treatment** **methods**	Curettage alone Curettage + Burr / Cautery	39 (31.97) 15 (11.03)	83 (68.03) 121 (88.97)	**< 0.001**	0.662

* Adjacency to the physis is only evaluated in patients with an open physis (*n = 162*).

** Chi-square and Fisher’s exact tests were used to compare categorical variables between groups. Kaplan-Meier analysis was performed for recurrence-free survival. Differences were considered statistically significant for p value < 0.05.

For the treatment methods, curettage was performed in all patients. Of these, 122 (47.29%) underwent curettage alone, while 136 (52.71%) received adjuvant treatments. Specifically, 20 (7.75%) underwent burring, 29 (11.24%) underwent cauterization, and 87 (33.72%) received both burring and cauterization in addition to curettage.

For the reconstruction, grafts were used in 88.33%, cement in 16.67%, and both in 5.00%. Allografts were used in 61.64%, autografts in 22.01%, and both in 26.35%. Fixation was needed in 18 patients: 16 with pathologic fractures and two with high risk of a pathologic fracture. Fixation was performed with plates and screws in six patients, with intramedullary nails in two patients, with posterior instrumentation in two patients, with Kirchner wires in five patients, and with Steinman in one patient. one patient required endoprosthetic reconstruction.

The median disease-free survival was ten months, and the time to first recurrence was 24.22 ± 22.14 months on average. Recurrence was more common for patients younger than five years (34.38% vs. 19.03%, *p* = 0.046) and in the case of a pathologic fracture (40.00% vs. 18.42%, *p* = 0.006). Moreover, recurrence was less common with the addition of burring and/or cauterisation to curettage (31.97% vs. 11.03%, *p* < 0.001). The presence of soft tissue edema or fluid-fluid levels in MRI sections was also investigated in relation to recurrence. While these findings did not significantly impact the recurrence rate, the time until recurrence was significantly shorter in cases with soft tissue oedema (median 5 vs. 12 months, *p* = 0.010) or fluid-fluid levels (median 6 vs. 12 months, *p* = 0.038).

The relationship between growth plate status and recurrence was also investigated. Out of 258 patients, the physis was open in 162 (62.79%). A total of 86 (33.33%) lesions were adjacent to the physis, while 76 (29.46%) lesions were adjacent to the joints. No significant difference in recurrence rates was observed between lesions adjacent to the physis and those non-adjacent to the physis (23.26% vs. 21.05%, *p* = 0.736). There was also no significant difference in recurrence rates between lesions adjacent to the joint and those not adjacent to the joint (18.42% vs. 22.47%, *p* = 0.47). Factors affecting recurrence were summarised in Table 1.

## Discussion

The most commonly reported symptoms of ABCs are pain, swelling, a palpable mass, pathological fractures, and limping [6, 7, 20, 21]. Our study found that pain was the most frequent symptom, consistent with the literature, occurring in 79.07% of cases. However, despite pain being the most common symptom in patients under 5 years of age, the number of patients presenting with pathological fractures was significantly higher compared to other age groups. This suggests that ABCs may have a shorter doubling time in younger individuals, emphasising the need for early diagnosis and treatment in this age group. Alternatively, younger children may be less effective in verbalising pain, especially in the early stages of the disease.

There are various treatment methods available for ABCs. The primary treatment method has been curettage with or without grafting [7, 9, 10, 19, 20]. In addition, adjuvant methods such as burring, electrocauterization, cryotherapy, argon laser coagulation, phenol, cement, hydrogen peroxide, and embolisation are available [4, 11, 12, 22]. Sclerotherapy using substances like polidocanol and doxycycline is also employed [13–15, 23]. In our study, electrocauterization and/or high-speed burring was applied in 52.7% of patients as an adjuvant treatment method to enhance the effectiveness of curettage. The purpose of high-speed burring is to augment curettage by mechanically disrupting the lesion down to the surrounding healthy bone. Similarly, electrocauterization aims to kill residual tumour cells through the heat effect [4, 7, 24, 25]. Various adjuvant treatment methods have evolved to reduce recurrence; however, there is no consensus in the literature on their exact effects on the recurrence rate.

In the study by Wang et al. [18], which included 31 patients, a recurrence rate of 3.2% were observed following the use of curettage, bone grafting, and burring. In a case series of 40 patients, Gibbs et al. [25], reported local control rates of up to 90% were achieved in patients who underwent curettage and high-speed burring. In another study by Döring et al. [26], which included 90 patients with primary ABC, it was concluded that burring after curettage resulted in superior recurrence-free survival compared to curettage alone. Consistent with these studies, our study found that the recurrence rate was lower in the patient group that received electrocauterization and/or burring as an adjuvant treatment compared to the curettage-alone group (11.03% vs. 31.97%, *p* < 0.001). On the other hand, there were studies indicating that adjuvant treatment methods have no significant effect on the recurrence rate. Lin et al. [27] concluded in their study that adding burr to curettage did not affect recurrence. In the study by Gettleman et al. [17], adjuvant treatment methods such as high-speed burr, coagulation, liquid nitrogen, and/or hydrogen peroxide were used in 70.5% of cases (91 out of 129). They reported that there was no significant difference in recurrence rate between the groups that received and did not receive adjuvant treatment. In another study, Levanon et al. [28], reported that cryoablation, as an adjuvant treatment method, had no significant effect on the recurrence rate. In this study, the group that underwent curettage and burring was compared with the group that received additional cryoablation, with recurrence rates reported as 9.1% vs. 7.1%, respectively (*p* = 0.553). However, this study did not include a curettage-alone group, and all patients underwent burring. The patient group that underwent curettage and burring in the study by Levanon et al. appears to have similar recurrence rates to the group in our study that underwent burring and/or electrocauterization after curettage (9.1% vs. 11.03%). Differences among studies in the literature may be due to variations in the follow-up durations, differences in the sizes of study groups, the implementation of surgical techniques, and differences between surgeons.

Several authors have documented a higher rate of tumour recurrence in younger age groups [17, 27, 29]. We found a significantly higher recurrence rate in patients under five years of age, which was consistent with previous series. Various explanations have been proposed for this situation. One possibility is that the clinical and pathological behaviour of ABCs in younger patients is more aggressive. Another factor contributing to the high recurrence rate may be surgeons’ excessive caution in preventing growth arrest in very young patients [17, 27, 30]. Some authors have also theorized that the increased biological activity of growth plates raises the risk of recurrence in young patients [25, 31]. However, our study found no significant relationship between physeal status or physeal contact and recurrence. In terms of findings on this topic, our study aligns with the results of Gettleman et al. [17]. They documented that patients aged six years or younger had an increased recurrence rate; however, no significant association was found with physeal contact.

**Fig. 5:**
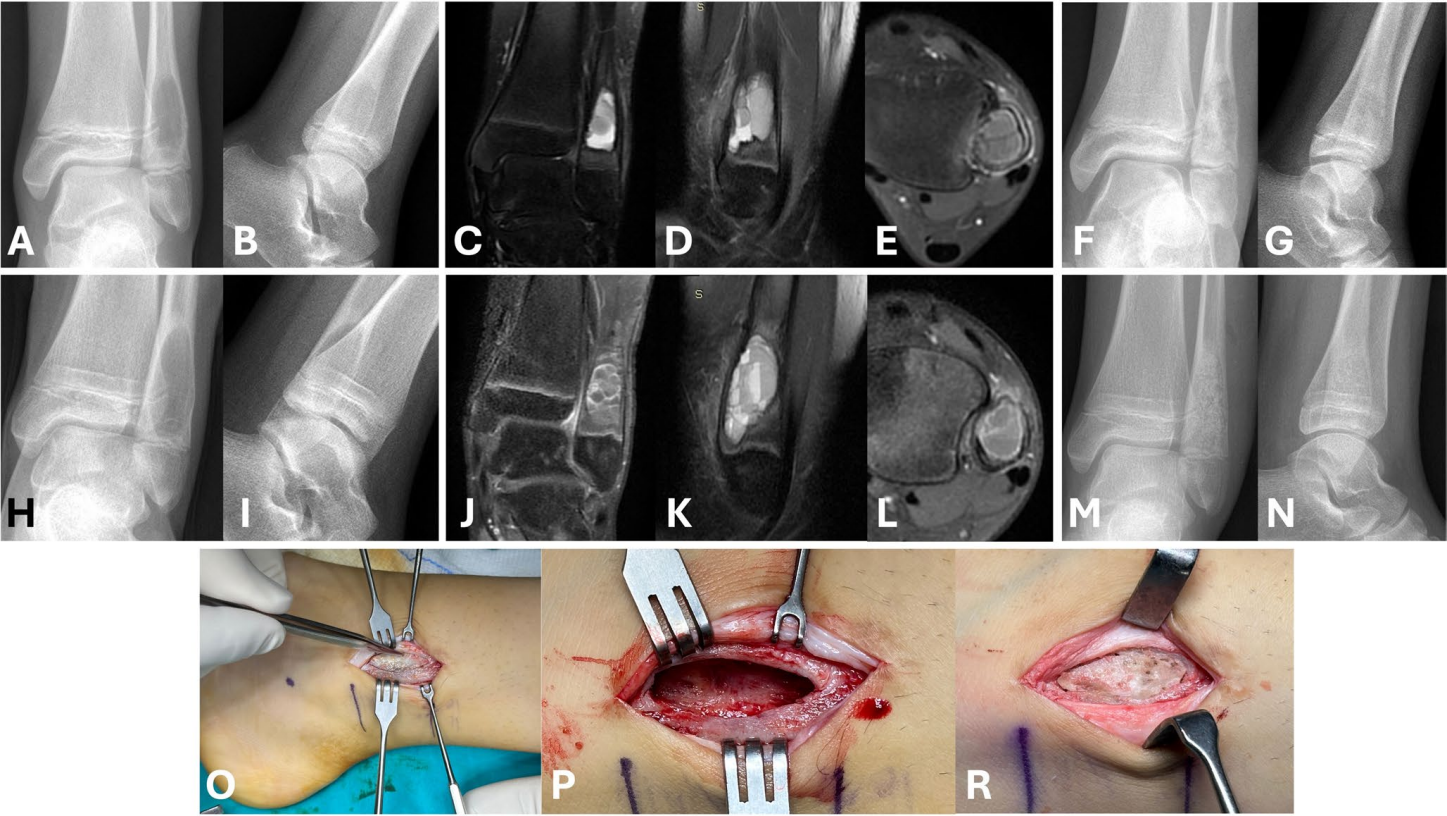
Illustration of the medical and surgical history of a 12-year-old female patient with a distal fibular aneurysmal bone cyst (**ABC**) and a recurrence in the postoperative first year (**A** & **B**) depict preoperative anteroposterior and lateral X-rays revealing a metaphyseal lesion in the distal fibula. (**C**, **D** & **E**) show preoperative MRI images highlighting the fluid-fluid levels characteristic of ABC. (**F** & **G**) display postoperative anteroposterior and lateral X-rays demonstrating the lesion filled with graft material following the initial surgery, which included curettage and cauterization. (**H** & **I**) present anteroposterior and lateral X-rays indicating the recurrence of the lesion. (**J**, **K** & **L**) provide preoperative MRI images confirming the recurrence. (**M** & **N**) show postoperative anteroposterior and lateral X-rays after the second surgery, which involved curettage, burring, and cauterization, and again depict the lesion filled with graft material. (**O**, **P** & **R**) include intraoperative images showing the bone lid, the space after curettage, burr and cauterization, and the reapplication of the bone lid after grafting

Fluid-fluid levels (FFL) within a bony lesion were first reported as a feature of aneurysmal bone cyst by Hudson et al. [32, 33]. FFL occur when fluids of different densities accumulate within a cystic structure [33, 34]. Although aneurysmal bone cysts and telangiectatic osteosarcoma are the most common bone lesions with fluid-fluid levels, these levels can be found in various bone and soft tissue lesions [33, 35]. Liquefaction due to tumour necrosis and intra-tumoral haemorrhage are possible mechanisms in the pathophysiology of FFL formation [33]. Van Dyck et al. reported FFL in 37% of ABCs in their series [34]. Davies et al. documented that 84% of ABCs contained FFL [36]. Our findings are consistent with the literature, as we observed FFL in 68.9% of our series. One noteworthy finding in our study is the shorter time to recurrence in the presence of soft tissue edema and fluid-fluid levels. Figure 5 shows an example case with these specific radiologic findings. Currently, no publication in the literature supports our findings regarding the relationship between recurrence time and the presence of FFL. The first possible reason may be that in lesions containing FFL, intraoperative bleeding control is more challenging, which may reduce the effectiveness of the applied curettage. The second possible reason is that ABCs with FFL may exhibit a more aggressive pattern. Evaluating this issue histopathologically in larger patient groups would provide valuable insights.

The study has several limitations. As a retrospective analysis, it lacks access to the specific criteria used for intraoperative decisions such as curettage, cauterisation, burring, grafts, cement, and fixation. Additionally, the study does not include sclerotherapy, preventing an assessment of its impact on recurrence in ABC treatment. Furthermore, the absence of USP6 and CDH11 oncogenes, specific to primary ABCs, limits diagnostic accuracy and differentiation [37]. Lastly, given the extensive 30-year period covered, changes in diagnostic and treatment practices over time may affect the evaluation of early versus late cases.

The study benefits from several notable strengths. It includes a large sample size, which enhances the reliability of the results. Patient records are well-maintained and highly accessible, contributing to the robustness of the data. Additionally, all surgeries were performed by expert senior surgeons at a tertiary centre, ensuring high-quality procedural standards. The study also spans a 30-year period, providing valuable insights into long-term outcomes and the evolution of diagnostic and treatment strategies.

## Conclusion

In conclusion, this study finds that aneurysmal bone cysts in patients under the age of five and those with pathological fractures are at a higher risk for recurrence. Electrocauterization or high speed burring as local adjuvant therapy leads to low recurrence rates. Therefore, early diagnosis—preferably before the occurrence of fractures—and effective local treatment are crucial for preventing recurrence.

## Data Availability

The data that support the findings of this study are available from the corresponding author upon reasonable request.

## Declarations

### Use of AI

Artificial intelligence (AI) tools were used solely for language editing purposes in the preparation of this manuscript. No AI tools were employed for data analysis, study design, or interpretation of results.
